# An unbiased proteomic analysis of PAD4 in human monocytes: novel substrates, binding partners and subcellular localizations

**DOI:** 10.1098/rstb.2022.0477

**Published:** 2023-11-20

**Authors:** Mekha A. Thomas, Seok-Young Kim, Ashley M. Curran, Barbara Smith, Brendan Antiochos, Chan Hyun Na, Erika Darrah

**Affiliations:** ^1^ Division of Rheumatology, Department of Medicine, School of Medicine, Johns Hopkins University, 5200 Eastern Ave, Suite 5200, Baltimore, MD 21224, USA; ^2^ Department of Neurology, Institute for Cell Engineering, School of Medicine, Johns Hopkins University, Baltimore, MD 21205, USA; ^3^ Department of Cell Biology, Institute for Basic Biomedical Sciences, School of Medicine, Johns Hopkins University, Baltimore, MD 21205, USA

**Keywords:** peptidylarginine deiminase, monocytes, citrullination, organelles, citrullinome, trafficking

## Abstract

Peptidylarginine deiminase IV (PAD4) post-translationally converts arginine residues in proteins to citrullines and is implicated in playing a central role in the pathogenesis of several diseases. Although PAD4 was historically thought to be a nuclear enzyme, recent evidence has revealed a more complex localization of PAD4 with evidence of additional cytosolic and cell surface localization and activity. However, the mechanisms by which PAD4, which lacks conventional secretory signal sequences, traffics to extranuclear localizations are unknown. In this study, we show that PAD4 was enriched in the organelle fraction of monocytes with evidence of citrullination of organelle proteins. We also demonstrated that PAD4 can bind to several cytosolic, nuclear and organelle proteins that may serve as binding partners for PAD4 to traffic intracellularly. Additionally, cell surface expression of PAD4 increased with monocyte differentiation into monocyte-derived dendritic cells and co-localized with several endocytic/autophagic and conventional secretory pathway markers, implicating the use of these pathways by PAD4 to traffic within the cell. Our results suggest that PAD4 is expressed in multiple subcellular localizations and may play previously unappreciated roles in physiological and pathological conditions.

This article is part of the Theo Murphy meeting issue ‘The virtues and vices of protein citrullination’.

## 1. Introduction

Peptidylarginine deiminases (PADs) have been implicated in playing a key role in several diseases including sepsis, thrombosis, cancer and autoimmunity [1,2]. Their pathogenic role is perhaps best understood in the context of rheumatoid arthritis (RA), a common systemic autoimmune disease characterized by chronic inflammation and irreversible damage to synovial joints [3]. A hallmark characteristic of RA is the presence of anti-citrullinated protein antibodies (ACPAs), which target proteins in which arginine residues have been post-translationally deiminated by PAD enzymes in a process known as citrullination. Of the five PAD isoforms (PAD1–4, and 6), PAD4 is expressed primarily by granulocytes and monocytes and is present at high levels in the inflamed RA joint [4–9]. Polymorphisms in the *PADI4* gene have been associated with RA development, and knocking out or inhibiting PAD4 has been shown to ameliorate disease in mouse models of RA [10–12]. While the importance of PAD4 to RA pathogenesis is clear, the subcellular localization and functions of the enzyme during normal physiology, which may be exploited in pathogenic states, remain understudied.

Although PAD4 was originally thought to be a nuclear protein due to the presence of a nuclear localization signal, new evidence suggests a more nuanced cellular localization [5]. More recent studies have also described PAD4 in the neutrophil cytosol as well as on the cell surface of neutrophils and monocytes, and importantly, have identified citrullinated proteins at these locations [13,14]. Additionally, the list of known PAD4 substrates has been expanding to include proteins from several subcellular locations including nuclear, cytosolic, cell surface and extracellular domains [15–20]. Importantly, however, it remains unknown how PAD4 traffics to and functions in extranuclear locations. In this study, we conducted an unbiased proteomic investigation of the cellular localization, organelle substrates and binding partners of PAD4, and identified monocyte differentiation into dendritic cells as a cellular system to study PAD4 trafficking. This systematic analysis revealed a previously undescribed subcellular localization of PAD4 to vesicular organelles and identified numerous novel substrates and putative binding partners involved in vesicular trafficking, suggesting a multi-faceted role for PAD4 in both health and disease.

## 2. Methods

### (a) Human subjects

De-identified leukopaks from healthy donors who gave platelets for medical purposes at the Anne Arundel Blood Donation Center were used as a source of leucocytes.

### (b) Monocyte isolation

Peripheral blood mononuclear cells (PBMCs) were separated using Ficoll-Pacque (GE Healthcare) density gradient centrifugation. Monocytes from the PBMC fraction were positively isolated using human CD14 Microbeads (Miltenyi Biotec) according to the manufacturer's instructions. Negatively isolated monocytes using the EasySep Human Monocyte Isolation Kit (STEMCELL Technologies) were used for transmission electron microscopy.

### (c) Monocyte subcellular fractionation and Western blot

5 × 10^7^ positively isolated monocytes were fractionated into subcellular compartments using the Minute Plasma Membrane Protein Isolation and Cell Fractionation Kit (Invent Biotechnologies) according to the manufacturer's instructions. Total cell lysates were created using 1 × 10^7^ monocytes lysed in NP40 lysis buffer supplemented with PMSF and protease inhibitors. Nuclear lysates were created in RIPA buffer supplemented with PMSF and protease inhibitors. Plasma membrane and organelle fractions were saved in NP40 lysis buffer with PMSF and protease inhibitors. Lysate protein concentrations were determined using bicinchoninic acid (BCA) assay (Pierce BCA Protein Assay Kit, Thermofisher) by following the manufacturer's protocol, and absorbance at 560 nm was read using Wallac Victor 3 1420 Multilabel Counter (PerkinElmer). 5 µg of protein were denatured in Laemmli buffer, loaded per condition and separated on 4–12% Bis-Tris SDS-PAGE gels (Invitrogen) at 200 V for 30 min. Proteins were transferred onto nitrocellulose membranes and immunoblotted using rabbit anti-human PAD4 (1:1000; N-terminal P4749, Sigma), rabbit anti-human Na^+^/K^+^ ATPase 1 (1 : 1000; 3010S, Cell Signaling), rabbit anti-human α-tubulin (1 : 1000, ab52866, Abcam), rabbit anti-human GAPDH (1 : 2000; 51745, Cell Signaling) and mouse anti-human lamin-B1 (1 : 500; ab8982, Abcam). Images of blots were taken using the FluorChem M detection system (ProteinSimple) and PAD4 levels were quantified using AlphaView SA software (ProteinSimple).

To quantify PAD4 and PAD2 levels, monocytes were positively isolated from peripheral blood from three healthy controls and total cell lysate in NP40 lysis buffer was prepared and quantified as described above. Lysates (5 µg) were resolved using SDS-PAGE and immunoblotted using mouse anti-PAD4 antibody (1 : 1000, ab128086, Abcam), rabbit anti-PAD2 antibody (1 : 1000; ab16478, Abcam) and mouse anti-β-actin antibody (1 : 1000; clone: AC-15, MilliporeSigma). Following HRP-labelled secondary antibody incubation, blots were imaged as mentioned above and protein levels were quantified using AlphaView SA software (ProteinSimple). PAD4 and PAD2 levels were normalized to β-actin levels present in each donor and graphed using GraphPad Prism 10.0.1 (Dotmatics).

### (d) Transmission electron microscopy

5 × 10^7^ negatively isolated monocytes using the EasySep Human Monocytes Isolation Kit (STEMCELL Technologies) were fixed using 4% paraformaldehyde, 0.1% glutaraldehyde, 3 mM MgCl2 in 0.1 M Sorenson's sodium phosphate buffer (pH 7.2) overnight at 4°C. Following a buffer wash, 1% osmium tetroxide, 0.8% potassium ferrocyanide in 0.1 M sodium phosphate was used to postfix samples for one to two hours on ice in the dark. 0.1 M maleate buffer was used to rinse samples after osmium treatment. The cells were stained en bloc using 2% uranyl acetate in maleate buffer for one hour in the dark, dehydrated in a graded series of ethanol and embedded in Eponate 112 (Polyscience) resin. Sample polymerization was conducted overnight at 60°C.

Sections measuring 60–90 nm were cut using a diamond knife on a Leica UCT ultramicrotome and picked up with Formvar coated 200 mesh nickel grids. To label sections with PAD4 antibodies, grids were hydrated using dH_2_O and floated for the rest of the steps. 1.5% sodium meta periodate was used to etch samples, which were subsequently rinsed using dH_2_O and 10 mM NH_4_Cl in Tris Buffered Saline (TBS). Samples were blocked using 2% BSA in TBS for 30 mins and incubated in primary antibody (1 : 25 dilution for C-terminal antibody (generated in-house [21]) and P4749 N-terminal antibody [Sigma], 1 : 50 for clone OTI4H5 [Abcam], or buffer) overnight at 4°C.

Following overnight incubation, grids were allowed to warm to room temperature for one hour and then rinsed with blocking buffer and TBS. Secondary antibody was added at 1 : 40 dilution at room temperature for 2 h, and samples were fixed using glutaraldehyde in sodium cacodylate buffer. Samples were then stained with 2% uranyl acetate and rinsed in water. Grids were imaged using a Hitachi 7600 TEM at 80 kV. An AMT CCD XR80 (8-megapixel camera – side mount AMT XR80 – high-resolution high-speed camera) was used to capture images.

### (e) Preparing samples for mass spectrometry

5 × 10^7^ positively isolated monocytes from three healthy donors were incubated in 5 mM calcium in Hank's Balanced Salt Solution (HBSS) for 3 h at 37°C. Cells were subjected to subcellular fractionation as described above. Lysates of organelle fractions were treated to remove detergents via methanol–chloroform precipitation. They were subsequently reconstituted in lysis buffer containing 8 M urea, 10 mM tris(2-carboxyethyl)phosphine (TCEP) and 40 mM chloroacetamide (CAA) in 50 mM triethylammonium bicarbonate (TEAB). Proteins were reduced and alkylated at room temperature for 1 h. 8 M urea was diluted to 2 M via the addition of three volumes of 50 mM TEAB before digestion. Trypsin (sequencing grade modified trypsin, Promega) was used to digest proteins at 10 ng µl^−1^ (v/v) at 37°C overnight. Following digestion, peptides were acidified using 1% trifluoroacetic acid (TFA) and desalted using C_18_ StageTips. Peptides were eluted, vacuum-dried and stored at −80°C before mass spectrometry analysis.

### (f) Mass spectrometry analysis

An Orbitrap Fusion Lumos Tribrid mass spectrometer interfaced with an Ultimate-3000 RS LCnano nanoflow liquid chromatography system (Thermo Scientific) was used to analyse peptides. To identify proteins from mass spectrometry spectra, files were analysed using Proteome Discoverer (version 2.4.1.15, Thermo Scientific). Mass spectra results were searched against the human UniProt database (released in Jan. 2021) containing common contaminant proteins using SEQUEST HT algorithms. The following parameters were used for database search: (1) trypsin with a maximum of two missed cleavage sites; (2) 10 ppm precursor mass error tolerance; (3) 0.02 Da fragment mass error tolerance; (4) fixed modification: carbamidomethylation (+57.02146 Da) at cysteines; (5) variable modifications: oxidation at methionine (+15.99492 Da), deamidation at arginine, asparagine and glutamine (+0.98402 Da), protein N-terminus acetylation (+42.01057 Da), loss of methionine (−131.04049 Da) and methionine loss with acetylation (−89.02992 Da). Minimum and maximum peptide lengths were set at 6 and 35 amino acids, respectively. A 1% false discovery rate was used to filter proteins and peptides [22–24]. Data processing workflow and representative spectra are provided. The mass spectrometry proteomics data have been deposited in the ProteomeXchange Consortium via the PRIDE partner repository with the dataset identifier PXD044188 [25].

Peptide data obtained from mass spectrometry were filtered based on citrullination of arginines (excluding those occurring only at C-terminal arginine residues) and the first accession number listed was used to find the corresponding protein for downstream analysis. Fifteen proteins were excluded due to low confidence, and the abundances of the resulting 271 proteins were analysed for each donor. The 257 proteins shared by all three donors were subsequently analysed for subcellular localization using the UniProt database [26]. Accession numbers of three proteins were listed as ‘deleted’, so they were substituted with the first protein accession number in UniProt under the same gene name. Proteins were sorted based on the first designated localization listed under ‘Subcellular localization’ and ‘Gene Ontology (cellular component)’, and proteins predominantly associated with nuclei and cytosol were removed from analysis. The resulting list of 99 citrullinated proteins were then analysed for statistical overrepresentation compared to the human proteome using the PANTHER classification system [27,28]. Ten proteins were unable to be mapped by PANTHER, so their accession numbers were substituted for the first listed accession number in UniProt under the same gene name. Statistical significance was determined using Fisher's exact test with Benjamini-Hochberg false discovery rate correction. Thresholds were set at false discovery rate corrected *p* = 0.01 and ±2 fold enrichment.

### (g) Citrullination site amino acid sequence motif analysis

Potential PAD4 citrullination site sequence motifs were evaluated by aligning the P6–P6′ positions around each deiminated arginine (i.e. citrulline) detected in the peptides corresponding to the 271 citrullinated proteins identified in the subcellular organelle fractions by mass spectrometry. Citrullination sites containing fewer than six amino acids on either side of the citrulline were excluded from motif analysis. The frequency of each amino acid at each position in the citrullination site was assessed using pLogo v.1.2.0 (Schwartz Lab, University of Connecticut) to generate a probability logo to visualize enrichment of amino acids within the citrullination site [29]. Log-odds of the binomial probabilities for each amino acid at each position were used to plot amino acid residues in proportion to their respective frequencies. Residue frequencies were assessed for over- or under-representation at each position compared to the human proteome. Significance thresholds were set at *p* < 0.05, represented as log-odds of ± 3.64.

### (h) HuProt binding chip assay

To identify potential surface binding partners of PAD4, PAD4 protein—purified as previously described [21]—was labelled with the Alexa Fluor (AF) 555 Protein Labeling Kit as per the manufacturer's instructions (ThermoFisher). Fluorescently labelled PAD4 protein was incubated on a blocked HuProt protein microarray chip (CDI Laboratories) that contained over 16 000 GST-tagged proteins for 1 h at room temperature with shaking. Since calcium binding to PAD4 induces conformation changes and catalysis, the incubation was performed at increasing calcium concentrations (0, 0.2, and 2 mM). Binding to candidate proteins was visualized by fluorimetry and normalized to fluorescence obtained with a Cy5-labelled anti-GST antibody. An A-score was calculated for each normalized protein value, representing the difference in PAD4 binding to each candidate protein minus the array average divided by the standard deviation. Binding partners with an A-score of greater than 2 were considered to be putative binding partners for further analysis. The resulting list of putative PAD4 binding partners was then analysed for statistical overrepresentation compared to the human proteome using the PANTHER classification system [27,28]. Statistical significance was determined using Fisher's exact test with Benjamini–Hochberg false discovery rate correction. Thresholds were set at false discovery rate corrected *p* = 0.01 and ±2 fold enrichment.

### (i) Analysing binding of PAD4 to putative binding partners

To confirm binding of PAD4 to candidate binding partners, 500 ng of recombinant human (rh)-CEACAM1 (SinoBiologicals), rh-enolase (purified as described [30]), rh-nucleophosmin (purified as previously described [31]), rh-MNDA (purified as previously described [32]), or 12.5 ng rhPAD4 (generated in-house as previously described [21]) were coated per well in triplicates onto high-binding ELISA plates (Corning) overnight at 4°C. Wash steps were performed using 0.02% Tween-20 in PBS. Plates were blocked using 2% BSA in PBS for 1 h at 37°C, followed by serial dilution (2600 ng to 0 ng) of rh-PAD4 in 0.2 mM Ca^2+^ and 100 mM Tris-HCl (pH = 7.5) for 2.5 hrs at room temperature with shaking. Wells were subsequently incubated with 1 : 250 dilution of rabbit anti-PAD4 (clone: P4749, MilliporeSigma) for 2 h and then with 1 : 7500 dilution of HRP-labelled anti-rabbit secondary antibody for 1 h at room temperature with shaking. TMB substrate (KPL) was used to develop wells, and the reaction was stopped by the addition of 1 M HCl. A Wallac Victor 3 1420 Multilabel Counter plate reader (PerkinElmer) was used to read absorbances at 450 nm and 560 nm (background). Absorbance at 0 ng PAD4 was subtracted from each PAD4 concentration for each binding partner and the adjusted absorbance was normalized to anti-PAD4 antibody binding to 12.5 ng of rhPAD4. Binding affinity plots and *K*_d_ values were generated using the ‘One-site – total binding’ model in GraphPad Prism 10.0.1 (Dotmatic).

### (j) Monocyte derived-dendritic cell (mo-DC) differentiation

Monocytes isolated using positive selection were cryopreserved in 90% heat-inactivated FBS and 10% DMSO until use. Thawed cells were resuspended at 1 × 10^6^ cells ml^−1^ in Mo-DC Differentiation Medium containing granulocyte monocyte-colony stimulating factor (GM-CSF) and interleukin-4 (IL-4; Miltenyi Biotec) for three days. On the third day, cells were supplemented with equal volume of media and cultured for three more days for a total of six days. On the sixth day, cells were harvested, counted and used for downstream assays.

### (k) Confocal microscopy

Monocytes (day 0) and mo-DCs (day 3 and day 6) were resuspended at 10 × 10^6^ cells ml^−1^ in PBS. 100 µl of cells were added dropwise onto coverslips and allowed to adhere for a minimum of 30 mins in 1 ml PBS. Cells were subsequently fixed in 4% paraformaldehyde for 30 mins and washed three times using PBS. When indicated, cells were permeabilized by incubating coverslips in 100% acetone for 30 s. 5% BSA in PBS was used to block for 30 mins at room temperature. Following three rinses in PBS, primary antibody dilutions were created in 1% BSA in PBS and added to coverslips for 1 h at room temperature. The following primary antibody dilutions were used in this study: mouse anti-human HLA-DR (1 : 50; clone: L243, Biolegend), mouse anti-human Lamp1 (1 : 100; clone: eBioH4A3, Thermofisher), mouse anti-human Rab7 (1 : 50; clone: E907E, Cell Signaling), mouse anti-human Rab5a (1 : 100; clone: 2E8B11, Thermofisher), mouse anti-human KDEL (1 : 100; SPA-827, Stressgen Technologies) and rabbit anti-human PAD4 (1 : 100; in-house generated). As a positive control for protein colocalization, cells were stained using rabbit anti-CD11b (1 : 100; clone: EPR1344, Abcam) and mouse anti-CD18 (1 : 50; clone: MEM-148, Thermofisher) antibodies. Coverslips were rinsed in PBS and incubated with 1 : 200 dilution of AF488 goat anti-mouse IgG1 (Invitrogen) or IgG2a (Invitrogen), or AF594 Plus donkey anti-rabbit (Invitrogen) secondary antibodies for 30 min at room temperature. Following rinses in PBS and dH_2_O, coverslips were mounted using ProLong Gold AntiFade DAPI mounting medium (Thermofisher) and sealed. Samples were imaged using a Zeiss AxioObserver with 880-Quasar confocal module & Airyscan FAST module, and images were converted using ZEN microscopy software (Zeiss). For colocalization analysis, four to five random fields were captured at 20X magnification and colocalizations were quantified using Volocity version 7.0.0.

### (l) Flow cytometry

Treated and untreated cells were stained for viability using blue fluorescent reactive dye (Invitrogen). Cells were washed and blocked using human BD Fc block (BD Biosciences) in 3% BSA in PBS and stained with anti-CD11c-BV421 (clone: Bu15; Biolegend). In-house generated rabbit anti-human PAD4 antibody was fluorescently labelled using AF647 antibody labelling kit (Invitrogen) and used at 0.01 µg µl^−1^. Equal amounts of AF647-labelled rabbit IgG (3452S, Cell Signaling Technology) served as isotype control. Cells were incubated with labelled antibodies for 30 min at 4°C and flow cytometry was conducted at the Johns Hopkins Bayview Immunomics Core Facility using Cytek Aurora (Cytek Biosciences). Data were analysed using FCS Express 7 Research Edition (De Novo Software).

## 3. Results

### (a) PAD4 is present in vesicular organelles in monocytes

While PAD4 has been documented in the cytosol, plasma membrane and nucleus of both granulocytes and monocytes, the mechanism by which PAD4 traffics to these localizations is still unknown. To better understand the subcellular localizations of PAD4, we fractionated monocytes from three healthy donors into various subcellular compartments and immunoblotted for the presence of PAD4 (electronic supplementary material, figure S1*a*). Purity of the subcellular fractions was demonstrated by immunoblotting for markers predominantly found in these localizations.

**Figure 1.**
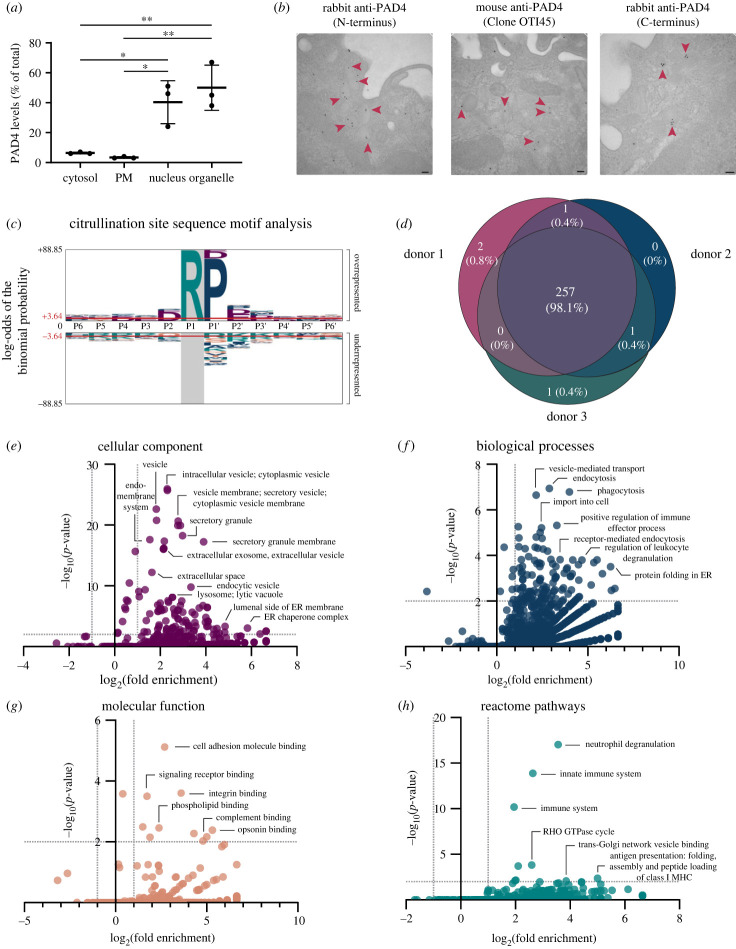
PAD4 is enriched in the organelle fraction in monocytes. (*a*) Monocytes isolated from peripheral blood of three healthy donors were subjected to subcellular fractionation and immunoblotted for PAD4. PAD4 levels were determined by dividing the amount of PAD4 expressed in a fraction by the sum of total PAD4 expression in all the subcellular fractions and compared using one-way ANOVA with Tukey's multiple comparisons test. Error bars represent mean ± standard deviation. PM, plasma membrane. (*b*) Monocytes isolated using negative selection from three healthy donors were stained for transmission electron microscopy using gold-labelled secondary antibodies against PAD4 targeting three distinct epitopes. Red arrowheads point to vesicular localizations of PAD4. Scale bars represent 100 nm. (*c*) PAD4 citrullination site amino acid sequence motif analysis (at positions P6–P6′) for all sites containing deiminated arginine residues detected by mass spectrometry in subcellular organelle fractions using pLogo. Log-odds equal to ± 3.64 represent a *p*-value < 0.05, and red lines indicate significance thresholds. The size of each letter is proportional to the log-odds binomial probability of that amino acid at its respective position, shown as over- or underrepresented at citrullination sites compared to the human proteome. (*d*) Venn-diagram of citrullinated organelle proteins identified by mass spectrometry on organelle fractions of monocytes (*n* = 3 donors) incubated in 5 mM calcium that were unique to or shared by donors. (*e–h*) PANTHER statistical overrepresentation analysis of citrullinated organelle proteins compared to the human proteome. log_2_ of fold enrichment versus –log_10_ of FDR-corrected *p*-values shown. Significance thresholds were set at ± 2 fold enrichment and a *p* < 0.01. Factors with fold enrichment greater than 0.01 are shown. *p < 0.05, **p ≤ 0.01.

PAD4 was detected in all cellular compartments including cytosol, plasma membrane, nucleus and organelles. While differences in PAD4 levels between the cytosol and the plasma membrane fractions were minimal, levels of PAD4 in the organelle fraction were significantly higher when compared to PAD4 levels in the cytosol (*p* = 0.0397) and the plasma membrane (*p* = 0.0350; figure 1*a*). The levels of PAD4 in the nuclear fraction were also significantly increased compared to the amount of PAD4 in the plasma membrane (*p* = 0.0446), as expected. However, there was no significant difference between PAD4 levels in the nuclear and the organelle fractions, indicating that organelles are a previously unappreciated reservoir for PAD4. To visualize the organelle localization of PAD4 in more detail, monocytes isolated from healthy donors were imaged via transmission electron microscopy using antibodies against three different epitopes of PAD4 (figure 1*b*, electronic supplementary material, figure S1*b*). Imaging revealed the frequent presence of PAD4 in vesicular organelles, with occasional staining observed in subcellular localizations suggestive of lysosomes, endoplasmic reticulum (ER) and mitochondria, regardless of the antibody used, confirming the enrichment of PAD4 in vesicular organelles (electronic supplementary material, figure S1*c*). Electron microscopy also confirmed a nuclear localization of PAD4 in monocytes, in accordance with previous studies (electronic supplementary material, figure S1*d*). Immunofluorescence of permeabilized monocytes further validated colocalization of PAD4 with lysosomes (Lamp1; average Pearson's coefficient = 0.5758; electronic supplementary material, figure S2*a* and *c*) and an even greater extent with the ER (KDEL; average Pearson's coefficient = 0.6595; electronic supplementary material, figure S2*b* and *c*).

### (b) PAD substrates are enriched in vesicular organelles and processes

To further elucidate the organelle localization of PAD4 and to examine whether citrullination of organelle proteins could be detected, monocytes from three healthy donors were incubated in calcium, fractionated and assessed for proteins in the isolated organelle fractions by mass spectrometry. We first searched the identified proteins for the presence of peptides derived from PAD4 or other PAD isoenzymes, since some studies have reported that monocytes also express PAD2 [8,33]. PAD4 peptides were identified in the organelle fraction of monocytes spanning 34% coverage of the PAD4 protein sequence (electronic supplementary material, database S1). Interestingly, PAD2 was the only other PAD family member identified, with peptides spanning 9% of the PAD2 sequence detected. PAD2 was slightly less abundant in organelles than PAD4, and this trend was confirmed in whole monocyte lysate by immunoblotting (electronic supplementary material, figure S2*d–f*).

Mass spectrometry results were subsequently filtered on proteins, with citrullination of arginines determined with high confidence (see workflow in electronic supplementary material, figure S2*g*; database S1). This analysis identified 271 proteins containing citrulline residues. Analysis of their citrullination site sequences revealed an overrepresentation of prolines in the P1′ (frequency = 43.53%, *p* = 3.54e-80) and P2′ (frequency = 12.30%, *p* = 0.006) sites and an enrichment of aspartic acid (P4, P2, P1′, and P2′ sites) and glutamic acid (P2′ and P3′ sites) residues surrounding the citrulline (figure 1*c*, electronic supplementary material, database S1). Of the 271 proteins found to be citrullinated, nine did not have an abundance above the detection limit and were discarded from subsequent analysis. Of the remaining 262 citrullinated proteins, 98.1% were shared by all three donors (figure 1*d*). Very few were donor-specific, with 0.8% (2/262) being unique to donor 1, 0% (0/262) found only in donor 2 and 0.4% (1/262) seen only in donor 3. The subcellular localizations of the 257 citrullinated organelle proteins shared by all three donors were then analysed using the UniProt database and proteins predominantly assigned to nuclear and cytosolic locations were excluded from subsequent analysis. Statistical enrichment analysis of citrullinated organelle proteins against the human proteome using the PANTHER database revealed multiple subcellular localizations of citrullinated proteins throughout various organelles (figure 1*e*, electronic supplementary material, database S1). Especially predominant were proteins belonging to vesicles and the endomembrane system, which parallels the observations made using electron microscopy (figure 1*b*). Proteins found in organelle membranes, mitochondria, vesicles, endosomes, and lysosomes were also significantly enriched. Interestingly, proteins found in the ER and Golgi were also citrullinated, suggesting an enrichment of PADs within components of the secretory pathway.

Analysing the enrichment of proteins participating in various biological processes revealed a dominant presence of citrullinated proteins involved in trafficking of vesicles, endocytosis, phagocytosis and protein folding in the ER (figure 1*f*, electronic supplementary material, database S1). Analysing citrullinated proteins involved in various molecular functions revealed a significant abundance of proteins involved in adhesion, especially those that participate in cell adhesion and integrin binding (figure 1*g*, electronic supplementary material, database S1). Intriguingly, proteins involved in signalling receptor, phospholipid, complement, and opsonin binding were also enriched, indicating citrullination of proteins trafficking to and present at or near vesicular and plasma membranes. Proteins involved in vesicle-mediated transport to various organelles, membrane trafficking and antigen presentation were also significantly enriched when analysing citrullinated proteins in reactome pathways (figure 1*h*, electronic supplementary material, database S1). Interestingly, neutrophil degranulation and pathways related to the innate immune system were also overrepresented. These data indicate that citrullination of several organelle proteins can be detected in monocytes, suggesting involvement of PADs in diverse cellular functions, with an enrichment in secretory pathway vesicles and functions.

### (c) PAD4 binds to protein partners found at various organelle compartments

The presence of PAD4 in vesicles and citrullination of proteins involved in secretory pathway organelles suggested that PAD4 may interact with protein binding partners in different subcellular locations to mediate its trafficking throughout the cell, possibly via an unconventional trafficking mechanism. Since PAD4 lacks conventional ER import signals and membrane-association domains, we hypothesized that PAD4 might be trafficking via binding to protein partners that escort it to various extranuclear compartments including secretory vesicles. To identify putative protein binding partners that may be involved in PAD4 trafficking, we analysed PAD4 binding to >10 000 human proteins using the HuProt protein microarray. Because PAD4 is a calcium-dependent enzyme, which may interact differentially with binding partners in the apo- or calcium-bound conformations [34], we probed for putative binding partners in the presence of 0, 0.2 and 2 mM calcium.

**Figure 2.**
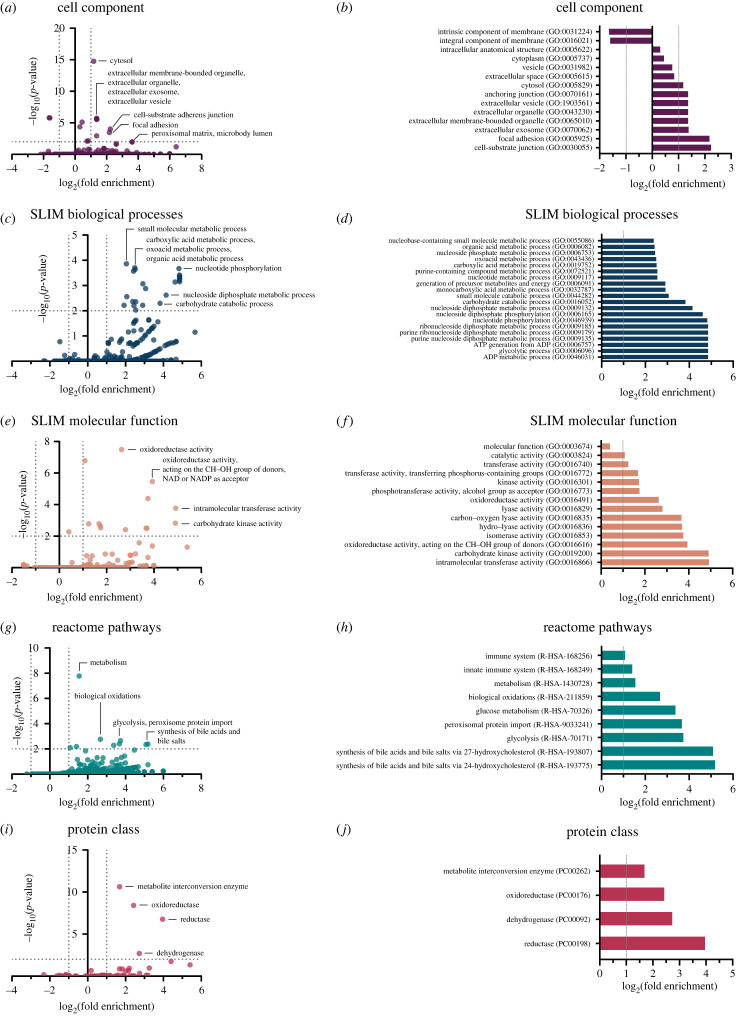
PAD4 binds protein partners found throughout the cell. PANTHER statistical overrepresentation analysis of PAD4 binding partners compared to the human proteome. (*a,c,e,g,i*) log_2_ of fold enrichment versus –log_10_ of FDR-corrected *p*-values shown. Significance thresholds were set at ±2-fold enrichment and a *p* < 0.01. Factors with fold enrichment greater than 0.01 are shown. Top twenty factors with *p* < 0 0.01 for (*b*) cell component, (*d*) SLIM biological processes, (*f*) SLIM molecular function, (*h*) reactome pathways and (*j*) protein class are shown. Fold enrichment cut-off values of ±2 are indicated by dotted grey lines.

Analysis of putative PAD4 protein binding partners, which bound with an A score greater than 2, revealed 234 candidates in 0 mM calcium, 280 candidates in 0.2 mM calcium and 298 candidates in 2 mM calcium (electronic supplementary material, figure S3*a*, database S2). Subsequent pathway analysis was then performed focusing on the 180 proteins that were bound in all calcium conditions (electronic supplementary material, figure S3*b*). Querying statistical overrepresentation of the 180 putative PAD4 binding partners in various cellular localizations compared to the entire human proteome revealed a significant enrichment of proteins found in the cytosol, extracellular environment, and cell junctions (figure 2*a*,*b*). Interestingly, proteins found in the peroxisomal matrix, microbody lumen and vesicles were also enriched, suggesting a trafficking mechanism via binding to transport proteins. Analysing statistical overrepresentation of biological processes revealed that proteins involved in metabolic processes—glycolysis in particular—were especially enriched as PAD4 binding partners (figure 2*c,d*). Proteins with several catalytic activities were also enriched as binding partners of PAD4 (figure 2*e,f*). This included proteins capable of kinase activity, transferase activity, lyase activity and oxidoreductase activity. Proteins involved in metabolic processes such as biological oxidations, glycolysis, peroxisome protein import and synthesis of bile acids were especially enriched in the reactome pathways analysis (figure 2*g,h*). Corroborating this evidence was the significant enrichment of metabolite interconversion enzymes, oxidoreductases, reductases and dehydrogenases as binding partners of PAD4 (figure 2*i,j*).

A deeper analysis revealed several known protein binding partners and substrates of PAD4, including NF-kB1, nucleophosmin (NPM1), and enolase (ENO1) [35–37], in addition to novel candidate surface binding partners with known expression on the monocyte surface including CEACAM1, OSMR and C5AR1 (electronic supplementary material, figure S3*c* and *d*) [38–40]. In addition, multiple Rab GTPase proteins involved in intracellular vesicular trafficking and signalling were identified as putative PAD4 binding partners, including RAB7L1, RABL3 and the Rab regulatory protein GDI2, as well as proteins involved in autophagy (ATG4C) and calcium-dependent membrane binding (CPNE2; electronic supplementary material, figure S3*e*) [41–43].

To validate the HuProt chip array findings, we determined affinities of PAD4 to a subset of proteins identified as putative binding partners *in vitro* using an ELISA. We found PAD4 to have increased affinity for nucleophosmin (*K*_d_ = 21.68), enolase (*K*_d_ = 27.74) and CEACAM1 (*K*_d_ = 79.50) compared to myeloid cell nuclear differentiation antigen (MNDA; *K*_d_ = 142.5), which was not found to interact with PAD4 on the HuProt array (electronic supplementary material, figure S3*f*). Together, these data suggest that PAD4 is capable of binding to diverse classes of proteins, with a preference for those with metabolic activity and transport functions across cellular compartments.

### (d) Monocyte-derived dendritic cells serve as a model to study cell surface PAD4 trafficking

**Figure 3.**
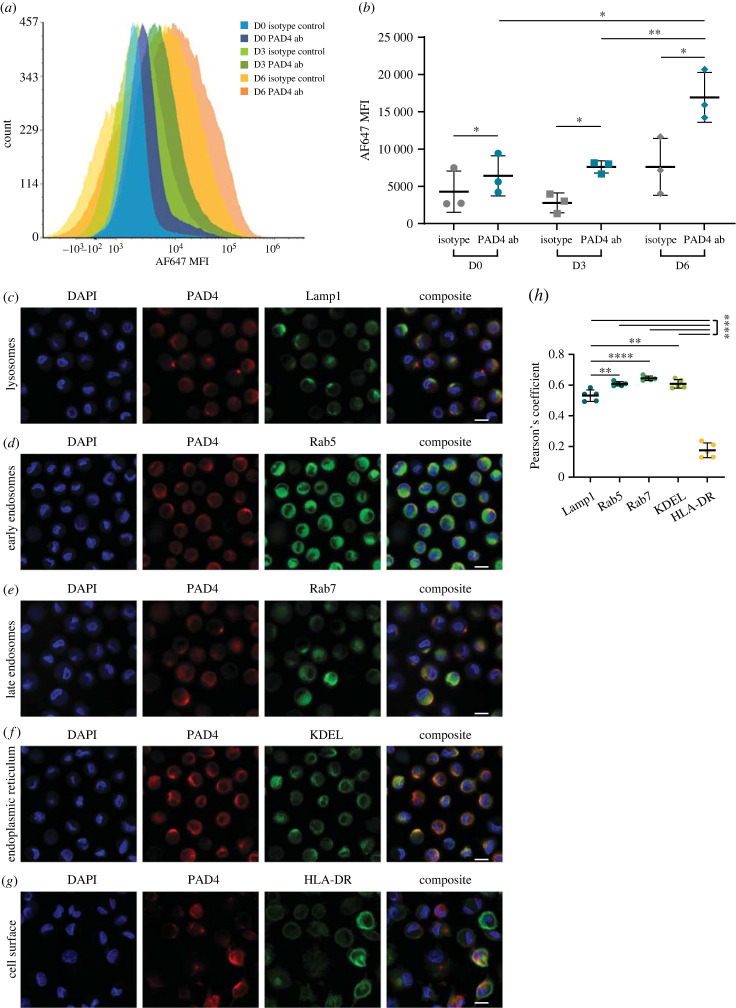
PAD4 associates with autophagic and exocytic organelles in mo-DCs. (*a,b*) Monocytes were differentiated into mo-DCs for six days and surface PAD4 levels were analysed using flow cytometry. (*a*) Representative histogram of surface PAD4 expression during mo-DC differentiation. (*b*) Quantitative analysis of mean fluorescence intensity (MFI) of isotype control (grey) or PAD4 antibody (blue) during mo-DC differentiation. MFIs between isotype control and PAD4 antibody were compared using paired *t*-tests, while MFIs of PAD4 antibody between mo-DCs of different culture days were compared via unpaired *t*-tests. Error bars represent mean ± standard deviation. (*c–g*) mo-DCs after six days of culture were allowed to adhere to coverslips and co-stained for nuclei (blue), PAD4 (red), and various organelle markers (green). Scale bars represent 10 µm. (*h*) Colocalization between PAD4 and organelle markers was analysed by averaging the Pearson's coefficients for five representative fields of view. Statistical analysis using one-way ANOVA and Tukey's multiple comparisons test was performed. Error bars represent mean ± standard deviation. *p < 0.05, **p ≤ 0.01, ****p ≤ 0.0001.

Because electron microscopy imaging and unbiased analysis of PAD4 substrates and binding partners revealed a putative role for PAD4 in intracellular vesicular compartments in monocytes, we used the cellular model of monocyte differentiation into mo-DCs to further understand the mechanisms involved in PAD4 trafficking. To first interrogate the surface expression of PAD4 during monocyte differentiation, monocytes were differentiated into mo-DCs for six days and the surface PAD4 expression was assessed using flow cytometry at days 0 (D0), 3 (D3) and 6 (D6). Increased surface expression of PAD4 during the differentiation period was observed with the greatest surface expression seen on D6 mo-DCs (figure 3*a,b*). Differentiation into mo-DCs was confirmed by the increased expression of CD11c (electronic supplementary material, figure S4*a*). Binding of the anti-PAD4 antibody to the cell surface was significantly higher than binding of the isotype control antibody to D0 monocytes (*p* = 0.0339), D3 mo-DCs (*p* = 0.0351) and D6 mo-DCs (*p* = 0.0215), indicating the presence of PAD4 on the surface of monocytes and mo-DCs (figure 3*a,b*). While D3 mo-DCs had similar levels of surface PAD4 to D0 monocytes (*p* = 0.5052), D6 mo-DCs had significantly higher expression of surface PAD4 (*p* = 0.0132 compared to D0 and *p* = 0.0093 compared to D3). Confocal imaging further revealed differences in PAD4 subcellular localization at different stages of monocyte differentiation to mo-DCs (electronic supplementary material, figure S4*b*). D0 monocytes and D3 mo-DCs had primarily extranuclear expression of PAD4. However, D6 mo-DCs had a highly polarized expression of PAD4 toward the cell surface, most commonly located apical to the nucleus.

Since D6 mo-DCs had the greatest expression of surface PAD4 and a polarized cellular distribution, they were used as a cellular system to further evaluate the mechanism of PAD4 trafficking, with a focus on evaluating secretory, endolysosomal and autophagy pathways [44]. Confocal imaging was performed to visualize and quantify colocalization of PAD4 with canonical endolysosomal markers in D6 mo-DCs using Pearson's correlation coefficient (figure 3*c–h*; electronic supplementary material, figure S4*c*, database S3). Pearson's correlation coefficient is a measure of the overlap between two markers of interest, with a coefficient of 1 indicating perfect overlap and values close to 0 suggesting no correlation between the two signals of interest [45]. Lamp1 staining, a marker of lysosomes, could be seen as large clusters present in D6 mo-DCs (figure 3*c*, electronic supplementary material, figure S4*c*). Although PAD4 and Lamp1 exhibited some colocalization (average Pearson's coefficient = 0.5314), cells with distinct localization of PAD4 or Lamp1 alone can also be seen (figure 3*c,h*, electronic supplementary material, figure S4*c*). Extranuclear localization of Rab5 (indicator of early endosomes and regulator of early autophagosome formation [44]) was seen to be dispersed throughout the cell, and PAD4 and Rab5 colocalized to a greater degree with an average Pearson's coefficient of 0.6086 (figure 3*d,h*, electronic supplementary material, figure S4*c*). Similar to Rab5, staining of Rab7 (a marker of late endosomes and meditator of autophagosome maturation [44]) was seen to be more similar to PAD4 staining, with the greatest average Pearson's coefficient of 0.6442 (figure 3*e,h*, electronic supplementary material, figure S4*c*). Interestingly, staining using KDEL to visualize proteins retained in the ER resulted in small punctate spots spread throughout the cytosol of mo-DCs (figure 3*f*, electronic supplementary material, figure S4*c*). Moderate colocalization of PAD4 and KDEL could also be seen, with a mean Pearson's coefficient of 0.6082 (figure 3*h*, electronic supplementary material, figure S4*c*). Strong cell surface and some intracellular compartmentalization staining could be seen with HLA-DR, however, the average Pearson's colocalization coefficient was only 0.1750, suggesting minimal colocalization with PAD4 (figure 3*g*,*h*, electronic supplementary material, figure S4*c*). Analysing the average Pearson's coefficients of all markers with PAD4 revealed significantly stronger colocalization of PAD4 with Rab5 (*p* = 0.0010), Rab7 (*p* < 0.0001), and KDEL (*p* = 0.0011) compared to Lamp1, and for all markers compared to HLA-DR *(p* < 0.0001). To confirm that these findings remained accurate while using conditions that permitted a more thorough cellular permeabilization, we repeated this analysis using PFA fixed and acetone permeabilized cells and found that the overall spatial relationship between PAD4 and markers of subcellular localizations remained largely unchanged, with increased Pearson's coefficients (electronic supplementary material, figure S5*a–g*, database S3). Taken together, our data indicate the presence of PAD4 in endolysosomal compartments involved in autophagy, as well as in conventional secretory compartments.

## 4. Discussion

While PAD4 has historically been categorized as a primarily nuclear enzyme, recent evidence has revealed its presence in other subcellular compartments including the cytosol and the cell surface [13,14]. A major question that remains unanswered is the mechanism by which PAD4, which contains a nuclear localization signal but lacks a canonical secretory signal sequence, traffics to the plasma membrane of neutrophils and monocytes [5]. In this comprehensive study, we observed that PAD4 associates with proteins found in a variety of subcellular localizations, with unexpected enrichment and activity in vesicular organelle compartments. We also found that PAD4 expression on the cell surface increases as monocytes differentiate into mo-DCs, and illuminated a probable trafficking mechanism via the endolysosomal and autophagic pathways.

Early studies conducted on PAD4 in granulocytes and transfected HeLa cells were the first to report the presence of a nuclear localization signal sequence and subsequent nuclear localization of PAD4 [5]. In support of this observation, various nuclear proteins have been documented to be subject to citrullination in a physiological environment, including histones [5,17,35,46,47]. However, the presence of PAD4 in the extranuclear space has been recently reported, with PAD4 additionally found in cytosolic and cell surface localizations [13,14]. We found that PAD4 is not only present in the cytosol, plasma membranes and nuclei of monocytes, but also in the organelle fractions in relatively large quantities. Surprisingly, we also found the presence of PAD2 in monocytes from the peripheral blood of healthy controls via mass spectrometry and Western blot. While PAD4 was known to be present in monocytes, protein expression of PAD2 in these cells was unclear, with discrepant reports regarding its expression [6,8,33]. Therefore, determining the localizations of PAD2 and its activity in monocytes should be subject to future studies to define the relative contributions of PAD2 and PAD4 to the organelle citrullinome in monocytes.

Our finding that PADs are capable of citrullinating various organelle proteins, including those involved in signalling, vesicular and protein trafficking, and a myriad of other biological processes, suggests that PADs may play a larger role in physiological monocyte cell function than previously appreciated. Our work also expands the human PAD-dependent citrullinome to include a large number of organelle proteins in addition to the cytosolic and nuclear proteins that were previously known. Understanding whether the citrullinated organelle proteins identified in this study are present in other subcellular localizations within monocytes and determining whether citrullination occurs at extranuclear and extracytosolic sites should be subject to further studies. Additionally, the effects of citrullination on the function and structure of organelle proteins and the role they play in healthy versus disease states need to be elucidated further in future studies.

PAD4 expression in the cytosol may be explained by the process whereby nuclear proteins are translated by free ribosomes present in the cytoplasm prior to nuclear import, and also by the fact that nuclear versus cytosolic distribution of proteins can be regulated by post-translational modifications [48,49]. However, the mechanism by which PAD4, which lacks ER import signal sequences and transmembrane domains, traffics to the cell surface and is retained there has been a mystery. While PAD4 is known to influence the function of cytoplasmic proteins (e.g. NADPH oxidase complex) and the translocation of nuclear proteins (e.g. Elk-1) [13,50], our data show that PAD4 may have a larger array of binding partners, which may influence its multiregional localization within the cell. We found, via an unbiased evaluation of organelle substrates and binding partners, that PAD4 can associate with proteins from several subcellular localizations, including the plasma membrane, cytosol, vesicles and cell junctions. Proteins participating in a wide range of metabolic activities, including glycolysis, were also statistically overrepresented as binding partners of PAD4, suggesting a possible novel role of PAD4 in regulating the metabolism in cells. Further work is needed to validate these putative binding partners and to evaluate how PAD4 influences the functional aspects of these proteins in physiological and pathological conditions.

Monocytes are a major infiltrating leucocyte in the rheumatoid joint and are high expressors of PAD4 with the capacity to differentiate into macrophages, osteoclasts and mo-DCs [51,52]. Mo-DCs play a key role in sampling the environment and presenting antigens to T cells at sites of inflammation, and previous studies had implicated associations between autophagy and PAD4-dependent citrullination in antigen-presenting cells [53,54]. Our findings show that surface PAD4 levels increase during mo-DC differentiation and that PAD4 distribution varies depending on the stage of differentiation. The colocalization of PAD4 with endolysosomal/autophagic vesicles and the ER implicates autophagy as well as the conventional secretory pathway, respectively, in PAD4 cellular trafficking. Importantly, the exact mechanisms of PAD4 association with the endomembrane system, the utilization of exocytosis and autophagy by PAD4 and implications of dysregulated PAD4 trafficking for the pathogenesis of diseases associated with citrullination, such as RA, need to be further explored.

Our study sought to use unbiased proteomic approaches to shed light on the complexities and nuances of PAD4 expression within monocytes and its implications for citrullination. Taken together, our results suggest that PAD4 has numerous potential binding partners, which it may utilize to traffic to various subcellular localizations. Citrullination of organelle proteins implies that PAD4 is active in these subcellular regions and is perhaps being held in an active conformation via association with its binding partner(s). PAD4 may then be exploiting conventional exocytosis/endocytosis pathways in an unconventional manner, in addition to autophagic processes, to traffic and modify substrates leading to citrullination of a large and diverse set of organelle-associated proteins. Understanding the role of PAD4 in subcellular compartments in healthy and disease states is an important next step in identifying novel targets for preventing aberrant PAD4 activation in diseases marked by dysregulated protein citrullination.

## Data Availability

All of the datasets supporting this article have been uploaded as part of the electronic supplementary material, databases S1–S3. The mass spectrometry proteomics data also have been deposited to the ProteomeXchange Consortium via the PRIDE partner repository with the dataset identifier PXD044188 [25]. The data are provided in electronic supplementary material [55].
